# Loss of Sorting Nexin 10 Accelerates KRAS-Induced Pancreatic Tumorigenesis

**DOI:** 10.1158/2767-9764.CRC-25-0168

**Published:** 2025-09-08

**Authors:** Kohinoor Khan, Mohammad Shameem, Ashley N. Sigafoos, Linda Kiey, Catherine E. Hagen, Gopal Ramakrishnan, Leslie Morse, Martin E. Fernandez-Zapico, Ricardo A. Battaglino

**Affiliations:** 1 Department of Family Medicine and Community Health, University of Minnesota, Minneapolis, Minnesota.; 2 Department of Rehabilitation Medicine, University of Minnesota, Minneapolis, Minnesota.; 3 Division of Oncology Research, Department of Oncology, Mayo Clinic, Rochester, Minnesota.; 4 Division of Anatomic Pathology, Department of Laboratory Medicine and Pathology, Mayo Clinic, Rochester, Minnesota.; 5 Targazyme Inc., Carlsbad, California.

## Abstract

Pancreatic ductal adenocarcinoma (PDAC), one of the most aggressive forms of pancreatic cancer, is associated with poor survival outcomes and currently ranks as the third leading cause of cancer-related death in the United States. Despite its clinical significance, the mechanisms of PDAC development and progression remain, in part, poorly understood. In this study, we provide evidence of a novel role of sorting nexin 10 (SNX10), a member of the sorting nexin family, in the regulation of KRAS-induced pancreatic carcinogenesis. We demonstrate that SNX10 is downregulated in PDAC, especially in advanced cases. Furthermore, mutational analysis revealed SNX10 genetic alterations in PDAC cases. Functional studies demonstrated that SNX10 overexpression in human PDAC cells inhibited cell proliferation and colony formation. Moreover, SNX10 overexpression induced G_1_-phase cell-cycle arrest and decreased KRAS signaling activity. Using a novel *Snx10* knockout mouse crossed with a *Kras*-driven PDAC model, we observed reduced survival, increased tumor cell proliferation, enhanced aggression, and heightened inflammation. Collectively, these findings highlight SNX10 as a tumor suppressor candidate in PDAC and underscore its promise as a foundation for new therapeutic approaches.

**Significance:**

SNX10 plays a crucial role in reducing pancreatic tumorigenesis. This discovery offers valuable insights into PDAC’s biology and the development of new effective treatments.

## Introduction

Pancreatic ductal adenocarcinoma (PDAC), the most frequent histologic subtype of pancreatic cancer, is a dismal condition, currently ranking as the third leading cause of cancer-related fatalities (1–3) with a 5-year survival rate of 13% (4). The PDAC mutational and epigenetic landscapes have been extensively described over the past three decades (5). However, our understanding of the mechanisms underlying PDAC pathobiology, particularly the effectors controlling KRAS-induced PDAC initiation, remains, in part, poor. This carries significant disease relevance, as KRAS is mutated in nearly 90% of PDAC cases, and it has been demonstrated to be sufficient to initiate PDAC development (6). Consequently, increasing the knowledge of the molecular events underlying KRAS oncogenic function is essential for developing more promising therapeutic targets for this dismal condition.

In this study, we define a novel role of sorting nexin 10 (SNX10) as a candidate tumor suppressor in PDAC. SNX10 belongs to a family of phosphoinositide-binding proteins participating in cargo sorting and protein trafficking along the endocytic pathway (7). Characterization of the *Snx10* knockout mouse model is essential for osteoclast formation and function (7). Moreover, we also illustrate that SNX10 interacts with PIKFYVE and FKBP12, key regulators of lysosomal biogenesis and vesicular trafficking (8, 9). Recently, we showed that SNX10 is essential for adipogenesis in human mesenchymal stem cells and is associated with waist-to-hip ratio adjusted for body mass index in women (10). In malignant disease, SNX10 has been reported to be downregulated in gastrointestinal malignancies, including PDAC, suggesting a potential tumor suppressor function (11–18). However, its role in cancer development remains underexplored. We have demonstrated that SNX10 expression decreases as PDAC progresses. Overexpression (OE) of SNX10 in human PDAC cells reduced cell proliferation. Furthermore, we found that SNX10 deficiency in genetically engineered mouse models promotes Kras-driven pancreatic tumorigenesis and reduces survival rates. Together, these findings establish SNX10 as a novel candidate tumor suppressor in PDAC, potentially antagonizing KRAS oncogenic function.

## Materials and Methods

### Cell culture

Human pancreatic cancer cell lines: AsPC-1 (RRID: CVCL_0152), HPAF-II (RRID: CVCL_0313), PANC1 (RRID: CVCL_0480), Panc 05.04 (RRID: CVCL_1637), MiaPaCa2 (RRID: CVCL_0428), HPAC (RRID: CVCL_3517), and BXPC3 (RRID: CVCL_0186), were purchased from the ATCC. AsPC-1, Panc 05.04, and BXPC3 were grown in RPMI 1640 media (ATCC, #30-2001). HPAF-II was cultured in Eagle minimum essential medium (ATCC, #30-2003). All cell lines were grown in a medium supplemented with 5% FBS (Cytiva, #SH30396.03) and 1% antibiotic (Gibco, #15140122). PANC1 and MiaPaCa2 were grown in DMEM (ATCC, #30-2002), supplemented with 5% FBS and 1% antibiotic. HPAC cells were grown in DMEM with high glucose and Ham’s F-12 medium (1:1 mixture; ATCC, #30-2006), supplemented with 0.002 mg/mL insulin (Gibco, #12585014), 0.005 mg/mL transferrin (Sigma-Aldrich, #T8158), 40 ng/mL hydrocortisone (STEMCELL Technologies, #74142), 10 ng/mL mouse EGF (STEMCELL Technologies, #78016.1), 5% FBS, and 1% antibiotic at 37°C in a humidified atmosphere of 5% CO_2_ (RRID:SCR_026100). All cell lines were authenticated by a short tandem repeat method. All cell lines were tested negative for *Mycoplasma* contamination using the MycoProbe Mycoplasma Detection Kit (R&D Systems, # CUL001B) at the beginning of each experiment, and cells were cultured for no more than eight passages for all the experiments.

### Generation of PDAC mouse models

All animal experiments were performed by and under the approval of the Institutional Animal Care and Use Committee at our institution. These studies were compliant with all federal and local guidelines. Mice of mixed background FVB/C57BL6 were used in animal studies. *Kras*^*G12D/+*^; *P48-Cre* (KC; RRID:MGI:2670727) and the *Kras*^*LSL.G12D/+*^; *Trp53*^*LSL.R172H/+*^; *P48-Cre* mice (KPC; RRID: IMSR_JAX:008179) have been previously described in detail (19, 20). The generation of *Snx10*^*fl/fl*^ mice was previously described in detail (21). In our study, we generated a pancreas-specific *Snx10* deletion by crossing P48Cre mice (RRID: IMSR_JAX:023329) with *Snx10*^*fl/fl*^ mice (RRID:IMSR_NM-CKO-226489) to generate *P48-Cre*; *Snx10*^*fl/fl*^(CS^fl/fl^) mice for studying the effect of *Snx10* loss alone. To assess tumor initiation, we created the *Kras*^*G12D/+*^; *P48Cre; Snx10*^*fl/fl*^ (KCS) model by crossing a male KC with a female CS^fl/fl^ mouse. For tumor progression analysis, we developed the *Kras*^*G12D/+*^; *P48Cre; Snx10*^*fl/fl*^; *Trp53*^*fl/+*^ (KPCS) model by crossing a male KPC with a female CS^fl/fl^ mouse. Although intermediate strains such as CS^fl/+^, KCS^fl/+^, and KPCS^fl/+^ were produced during breeding, only homozygous CS^fl/fl^, KCS^fl/fl^, and KPCS^fl/fl^ mice were included for further experimental study. To ensure accurate genotyping, all mouse lines used in this study were first validated through TransnetYX, Inc. using qPCR, a genotyping method that determines wild-type, heterozygous, and homozygous floxed alleles. Based on the genotyping results, 8- to 9-week-old healthy mice were included randomly in a mixture of males and females for all the experiments. A total of 10 to 14 mice were aged for survival, and Kaplan–Meier curves were generated per genotype. All mice included in the survival analysis were euthanized when they reached the endpoint or were 104 weeks old. The maximum tumor burden was limited to less than 2 cm in any direction, and this burden limit was not exceeded in the whole study. Pancreatic tumors were harvested and analyzed using gross pathology, histology, and molecular levels (mRNA and protein levels). Genomic DNA was also isolated from the pancreatic tissue to reconfirm the loss of genes in the mouse pancreas. Further recombination was confirmed by PCR for the final experimental strains. The wild-type *Snx10* was at 224 bp, the *Snx10*^*fl/fl*^ band was at 273 bp, the LSL-*Kras*^G12D^ bands were at 500 bp, and the *P53*^*fl/+*^ was at 612 bp. The recombination primers are listed in Supplementary Table S1.

### RNA extraction and RT-PCR

Total RNA was extracted from all PDAC cell lines and mouse tissues using TRIzol reagent (Invitrogen, #15596026), following the manufacturer’s protocol. The purity and integrity of total RNA were confirmed by NanoDrop [Thermo Scientific NanoDrop 2000c Spectrophotometer (RRID:SCR_020309)]. For cDNA synthesis, 1 μg of total RNA was reverse-transcribed using the high-capacity cDNA reverse transcription kit (Applied Biosystems, #4368814). Quantitative real-time PCR was performed on the QuantStudio 3 qPCR System (RRID:SCR_018712) using the TaqMan probes and fast advanced master mix (Applied Biosystems, #4444556). Thermal cycling parameters were as follows: 50°C for 2 minutes, with an initial denaturation step of 95°C for 20 seconds, followed by 40 cycles of denaturation at 95°C for 1 second, annealing, and elongation at 60°C for 20 seconds. The expression relative to β-actin or RPL13A was determined using the 2^−ΔΔCt^ comparative cycle threshold method to calculate the fold increase (22). For the human PDAC cell line, normal human pancreas RNA (Thermo Fisher Scientific, # QS0621) was used as a control compared with all PDAC cell lines. The primers are listed in Supplementary Table S1.

### Immunofluorescence

The unstained tissue microarray (TMA) slide was deparaffinized and rehydrated. For antigen retrieval, the slide was incubated in a 6.0 pH buffer (Reveal Decloaking reagent, Biocare Medical) for 30 minutes at 95°C to 98°C. Endogenous peroxidase activity was quenched by slide immersion in a 3% hydrogen peroxide/Tris buffered saline with Tween-20 (TBST) solution. The slide was incubated in a blocking solution for 30 minutes. Next, the slide was incubated in conjugated primary antibodies overnight at 4°C. The following day, the slide was washed with TBST and coverslipped using a diamond antifade mountant with DAPI (Invitrogen, #62248). The images were captured using a ZEISS Axio Imager M2 microscope (RRID:SCR_024706) and analyzed using ImageJ (RRID:SCR_003070), including parameters (Supplementary Table S2). The antibodies are listed in Supplementary Table S1.

### Tissue processing, histopathology, and IHC analysis

Freshly isolated pancreata are fixed in 4% paraformaldehyde overnight and stored in 70% ethanol. Fixed tissues were embedded in paraffin, sectioned (4 μm thick), and mounted on glass slides. For histopathology staining, slides were deparaffinized and hydrated. Slides were stained with Harris hematoxylin (made in BLS Histology Laboratory), rinsed in tap water, and dipped in ammonia water for bluing. The slides were counterstained with eosin Y (made in BLS Histology Laboratory) for 1 minute, dehydrated with 100% ethanol, and washed with xylene 3 times. After that, the slides were mounted with mounting media and coverslipped. Images were captured using a ZEISS Axio Imager M2 microscope (RRID:SCR_024706). Slides were blindly evaluated and scored by an expert pathologist (C.E. Hagen).

For IHC, antigen retrieval was performed by incubating slides in 6.0 pH citrate buffer (Reveal Decloaking reagent, Biocare Medical) for Ki-67 and F4/80 and EDTA 8.0 pH buffer for cytokeratin 19 (CK19) for 30 minutes at 95°C to 98°C. Endogenous peroxidase activity was quenched with a 3% hydrogen peroxide solution. Slides were blocked using Rodent Block M solution (Biocare Medical) for 30 minutes at room temperature (RT). Slides were incubated in primary antibody for 60 minutes at RT. After that, slides were washed with 1× TBST 3 times and incubated in a secondary antibody solution for 30 minutes at RT. All slides were washed with 1× TBST, incubated in avidin–biotin complex (Vector Laboratories), and detected with diaminobenzidine (BioLegend). Slides were counterstained with CAT Hematoxylin (Biocare Medical) for 5 minutes, dehydrated, and coverslipped. Images were captured using a ZEISS Axio Imager M2 microscope (RRID:SCR_024706). The antibodies are listed in Supplementary Table S1. For Masson’s trichrome staining, tissue sections were deparaffinized and hydrated in water. Sections were mordanted in preheated Bouin’s solution overnight at RT and then stained with Weigert’s iron hematoxylin for 10 minutes. After rinsing and washing, sections were stained with Biebrich scarlet–acid fuchsin for 2 minutes, followed by 15 minutes in phosphotungstic–phosphomolybdic acid solution. Slides were then stained with aniline blue for 5 minutes, treated with 1% acetic acid for 5 minutes, dehydrated sections, washed with xylene, and coverslipped using a synthetic mounting media. Images were captured using a ZEISS Axio Imager M2 microscope (RRID:SCR_024706). QuPath software (RRID:SCR_018257) was used to quantify the number of positive cells (nuclear brown signal) relative to the total number of cells for CK19, Ki-67, and F4/80 stains. For the Masson’s staining, the total blue signal of collagen over the entire tissue area was measured in each slide. Parameters applied for image analysis in QuPath are listed in Supplementary Table S2. Validation of the QuPath analysis was done blindly by an expert pathologist (C.E. Hagen).

### Protein extraction and Western blot analysis

Human PDAC cells or mouse tissues were lysed in 1× RIPA buffer (Thermo Fisher Scientific, #89900) supplemented with protease and phosphatase inhibitors (Thermo Fisher Scientific, #78440), incubated for 30 minutes on ice, and then centrifuged at 16,000 × *g* for 30 minutes at 4°C, as described previously (8). Total proteins were quantified using a Protein Assay Kit (Bio-Rad, #5000002) according to the manufacturer’s protocol. A total of 40 µg of protein was electrophoresed on a 4% to 15% gradient polyacrylamide gel (Bio-Rad, #4561084) with SDS and electroblotted onto polyvinylidene difluoride membranes (Thermo Fisher Scientific, #88520). Membranes were blocked in 1× TBST (Thermo Fisher Scientific, #28360) with 5% nonfat milk (Bio-Rad, #1706404) and probed with primary antibodies. Bound proteins were detected with horseradish peroxidase–conjugated secondary antibodies and SuperSignal West Dura Extended Duration substrate (Thermo Fisher Scientific, #34075) using the iBright CL1500 Imaging System (Invitrogen; RRID:SCR_026565). Bands were quantified using ImageJ software (RRID:SCR_003070). The antibodies are listed in Supplementary Table S1.

### SNX10 plasmid transfection, cell viability, and proliferation assay

AsPC1, HPAF-II, and PANC1 cells were transfected with 1 μg of SNX10 plasmid (NM_013322, Human Tagged ORF Clone, OriGene, cat. # RC206019; https://cdn.origene.com/datasheet/rc206019.pdf) and empty vector (OriGene; #pCMV6; RRID: Addgene_133867) using X-tremeGENE HP DNA transfection reagent (Sigma, #6366244001) and Opti-MEM (Thermo Fisher Scientific, #31985062) for 24 hours, following the manufacturer’s protocol. The cells were plated for further experiments.

For the MTT assay, 4 × 10^3^ cells/well were seeded in the 96-well plates after 24 hours of SNX10 transfection. Cell proliferation was measured at different time points (6, 24, 48, and 72 hours), as described previously (23).

For the cell growth assay, 3 × 10^3^ cells/well were seeded in the 12-well plates after 24 hours of SNX10 transfection. Cell growth was measured at different time points (days 1, 3, 5, and 7). At each time point, cells were trypsinized with 0.25% trypsin (Gibco, #25200072) and counted with the help of a hemocytometer (RRID:SCR_025846).

For the colony formation assay, 1,000 to 1,500 cells were plated into six-well plates and grown for 15 days. The colonies were fixed in methanol and acetic acid (1:1) for 20 minutes and then washed 3 times with 1× PBS. Fixed colonies were stained for 30 minutes with 0.5% crystal violet (Sigma-Aldrich, cat. # 548629) prepared in methanol. Colonies were counted after gently washing the dye with tap water and air-drying at RT.

For the cell-cycle assay, AsPC1 and HPAF-II (3 × 10^5^ cells/well) were seeded in six-well plates after 24 hours of SNX10 transfection. Cell-cycle analysis was performed at different time points: 24, 48, and 72 hours. The cells were collected by trypsinization, washed twice in 1× PBS, fixed with ice-cold 70% ethanol dropwise, and stored at −20°C. After fixation, the cells were washed with cold 1× PBS twice and resuspended in 500 μL of ready-to-use FxCycle propidium iodide (PI)/RNase staining solution (Thermo Fisher Scientific, #F10797) for 15 to 20 minutes at RT, protected from light. The cells were then run on a flow cytometer, BD FACSymphony A3 Cell Analyzer (RRID:SCR_023644). Data were analyzed using Floreada.io (January 30, 2025; RRID:SCR_025286).

### Human datasetsPI

The RNA sequencing data were downloaded from the Human Protein Atlas (RRID:SCR_006710) of patients with human PDAC [The Cancer Genome Atlas (TCGA) dataset, *n* = 176] and adjacent normal control tissues [The Genotype-Tissue Expression (GTEx) dataset, *n* = 328; refs. 24, 25]. The SNX10 genetic alterations in human PDAC were downloaded from the International Cancer Genome Consortium (ICGC; RRID:SCR_021722) and the Catalogue of Somatic Mutations in Cancer (COSMIC; RRID:SCR_002260). The following datasets with a total sample number are as follows: ICGC-PAAD-US, 185 samples (USA); ICGC-PACA-CA, 317 samples (Canada); ICGC-PACA-AU, 461 samples (Australia); and COSMIC, 7,337 samples (26, 27). The pie chart was generated using Canva (https://www.canva.com). Venn diagrams were created using Venny 2.1 (RRID:SCR_016561).

### Tissue microarrays

A TMA comprising 80 samples, including various stages of pancreatic cancer and normal pancreatic tissue, was obtained from TissueArray.com (PA804a). The array includes information on pathology grade, tumor–node–metastasis classification, and clinical stage. Of the 80 samples, 35 are from early-stage cases, 35 are from mid-to-advanced stage cases, and 10 are normal pancreatic tissues. Each case is represented by a single core.

### Quantification and statistical analysis

Statistical significance between two groups was determined using an unpaired Student *t* test, two-tailed unless otherwise indicated. For multiple group comparisons, a one- or two-way ANOVA was performed, and Bonferroni’s posttests were employed to determine statistical differences from the control group unless otherwise indicated in the figure legend. For the survival data, power analysis was conducted to determine the sample size. Kaplan–Meier curves were plotted and compared using a log-rank (Mantel–Cox) test, and a *, *P* < 0.05 was considered significant. Power analysis was performed using G*Power (RRID:SCR_013726). The statistical analysis and corresponding graphical representation were performed using GraphPad Prism 5 (RRID:SCR_002798). Cell-cycle data were analyzed by Floreada.io (RRID:SCR_025286). All the experiments were performed as a set of three repeats for validation and expressed as mean ± SEM, and *, *P* < 0.05; **, *P* < 0.01; and #, *P* < 0.001 were considered statistically significant.

### Data availability

This study used publicly available datasets from COSMIC (https://cancer.sanger.ac.uk/cosmic), ICGC (https://dcc.icgc.org), TCGA via Human Protein Atlas (https://www.proteinatlas.org/ENSG00000086300-SNX10/cancer/pancreatic+cancer#PAAD_TCGA), and the GTEx dataset (https://www.proteinatlas.org/ ENSG00000086300-SNX10/tissue/pancreas). All data were accessed under the respective data usage policies. The authors confirm that the data supporting the findings of this study are available within the article and its Supplementary Materials or from the corresponding author upon request.

## Results

### SNX10 genetic alterations and expression in human PDAC

**Figure 1:**
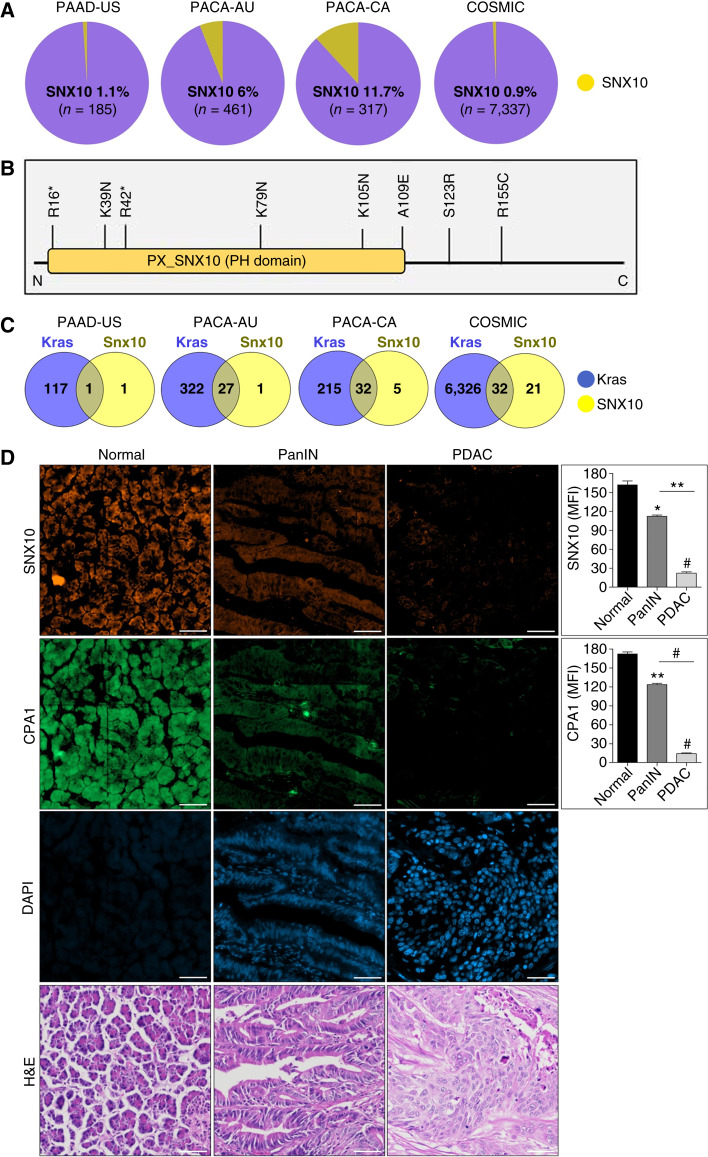
SNX10 genetic alterations and expression in human PDAC. **A,** Interrogation of PDAC from ICGC (https://dcc.icgc.org) and COSMIC (https://cancer.sanger.ac.uk/cosmic), showing mutations in the SNX10 gene. **B,** A schematic of the SNX10 gene shows the distribution of the mutations in the coding region (https://cancer.sanger.ac.uk/cosmic). **C,** Venn diagrams of SNX10 mutations that exist in conjunction with Kras gain-of-function alterations and overlap among SNX10 and Kras in PDAC (ICGC and COSMIC datasets; https://dcc.icgc.org/ and https://cancer.sanger.ac.uk/cosmic). **D,** TMA of normal, PanIN, and PDAC samples (left) stained with fluorescent IHC for CPA1 (green) and SNX10 (orange) expression. Scale bar, 20 μm. Quantification (right) of mean fluorescence intensity (MFI) of SNX10 and CPA1. Results indicate a progressive reduction in SNX10 and CPA1 expression from PanIN through advanced PDAC stages. Statistically significant differences *, *P* < 0.05; **, *P* < 0.01; and #, *P* < 0.001 are represented as mean ± SEM. H&E, hematoxylin and eosin; PH, Phox homology.

We initially performed a detailed analysis of PDAC from the ICGC and the COSMIC datasets for SNX10 mutations. We found that somatic mutations were present in PDAC in 0.5% to 11% of the cases, depending on the dataset (Fig. 1A). A significant proportion of these mutations, including nonsense, missense, and substitution missense, were present within the coding region, including the Phox homology domain, a region critical for the regulation of SNX10 activity (Fig. 1B; refs. 28, 29). Further analysis of the datasets revealed that a significant proportion of SNX10 mutations cooccurred with Kras gain-of-function alterations (Fig. 1C). RNA sequencing data using PDAC samples (TCGA dataset, *n* = 176) and adjacent normal control tissues (GTEx dataset, *n* = 328) revealed a significant reduction in SNX10 expression in PDAC tissue (Supplementary Fig. S1A). IHC staining data comprising sections from cancer TMAs, sourced from the Human Protein Atlas database, confirmed a progressive decline in Snx10 protein expression as cancer advanced compared with normal tissues (Supplementary Fig. S1B). Validation using TMAs containing cases from various stages of PDAC, pancreatic intraepithelial neoplasia (PanIN), and normal tissues showed a progressive reduction in SNX10 and carboxypeptidase A1 (CPA1) expression from PanIN through PDAC cases. CPA1 expression served as a positive control for normal cases (Fig. 1D). Finally, we examined SNX10 expression in human PDAC lines, AsPC-1, HPAF-II, PANC1, Panc05.04, MiaPaCa2, HPAC, and BXPC3 by qPCR. CPA1 and CK19 were used as positive controls for normal and tumor pancreas, respectively. Our results revealed that SNX10 is significantly decreased and CK19 expression is increased in all the PDAC cells compared with the normal pancreas (Supplementary Fig. S2A–S2C). Together, these results show the presence of mutations in key functional domains and reduced expression of SNX10, suggesting that this sorting may be relevant to the biology of PDAC.

### SNX10 OE suppresses cell proliferation

**Figure 2:**
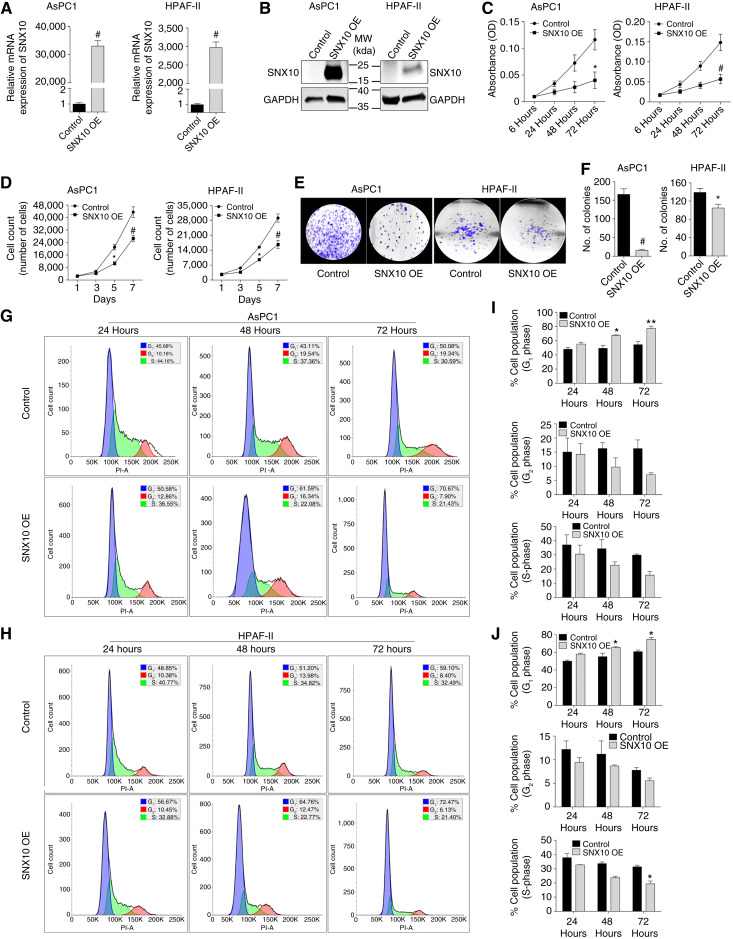
OE of SNX10 decreased cell proliferation in AsPC1 and HPAF-II PDAC cell lines. **A,** SNX10 transfection confirmation in AsPC1 and HPAF-II cells by qPCR fold change expression. Actin was used as a reference gene. **B,** Western blot of control vs. SNX10 OE with GAPDH was used as loading control for cell lines AsPC1 (left) and HPAF-II (right). **C,** Cell proliferation via MTT (absorbance at 570 nm) of AsPC1 (left) and HPAF-II (right) over 72 hours. **D,** The growth curve assay of AsPC1 (left) and HPAF-II (right) determined the growth rate over 7 days. **E,** Representative images of control vs. SNX10 OE colony-forming ability in AsPC1 and HPAF-II cell lines. **F,** Quantification of colony numbers in each group observed in **E**. Cell-cycle analysis of OE of SNX10 in (**G**) AsPC1 and (**H**) HPAF-II cells by flow cytometry shows that the percentage of cells in the G_1_ phase (blue) is higher than in the G_2_ phase (red) and S-phase (green) in the SNX10 OE group compared with the control. Cell-cycle data were analyzed using Floreada.io https://floreada.io/analysis. **I** and **J,** Quantification of cell-cycle analysis of the SNX10 OE group at all time points (24, 48, and 72 hours) compared with the control group in both AsPC1 and HPAF-II cell lines. Data are representative of three independent experiments. Statistically significant differences *, *P* < 0.05; **, *P* < 0.01; and #, *P* < 0.001 are represented as mean ± SEM. MW, molecular weight; OD, optical density; PI-A, propidium iodide area.

**Figure 3:**
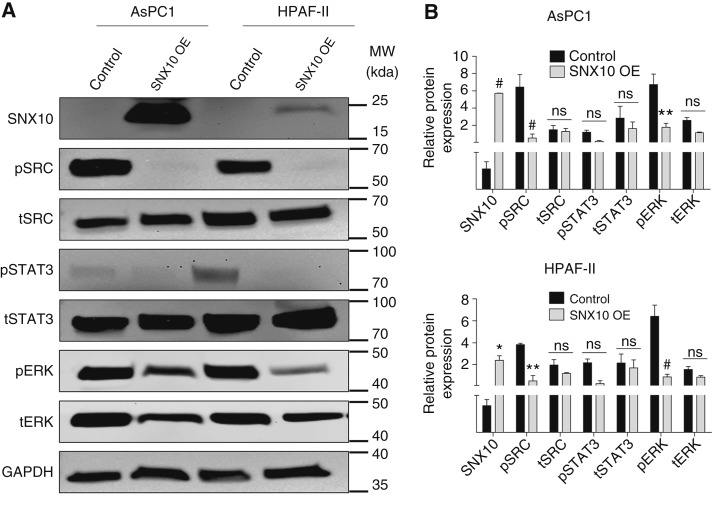
OE of SNX10 participated in regulating the protumorigenic protein expression. **A,** Western blot of control vs. SNX10 OE in AsPC1 and HPAF-II cell lines probing for the expression of protumorigenic markers. Protumorigenic markers probed for both total and phosphorylation of SRC, STAT3, and ERK expression and SNX10 (48 hours after treatment). Three independent western blots were performed, and the same blot was stripped for all the genes. **B,** Quantification of relative protein expression of genes in each group from **A**, with GAPDH used as a loading control. AsPC1 cell line is shown at the top, and HPAF-II at the bottom. Data are representative of three independent experiments. Statistically significant differences *, *P* < 0.05; **, *P* < 0.01; and #, *P* < 0.001 are represented as mean ± SEM. SNX10 OE was compared with the control group. MW, molecular weight.

Based on the above findings, we sought to evaluate the effect of SNX10 on cell growth in KRAS mutant PDAC cells AsPC1 and HPAF-II. These cells were transfected with the SNX10 plasmid for 24 hours and analyzed by MTT, cell counting, and colony formation assay. The levels of OE were confirmed by qPCR and Western blot (Fig. 2A and B), showing that the SNX10 OE group had increased expression compared with the control cells transfected with the empty plasmid (control). Interestingly, OE of SNX10 in AsPC1 and HPAF-II cells led to a significant reduction in cell proliferation at 72 hours (Fig. 2C), cell numbers at days 5 and 7 (Fig. 2D), and fewer colonies compared with the control groups (Fig. 2E and F). We also found that in AsPC1 (Fig. 2G and I) and HPAF-II cells (Fig. 2H and J), the SNX10 OE group resulted in a significantly higher percentage of cells in the G_1_ phase at the 48- and 72-hour time points, whereas there was a significantly lower percentage of cells in the S-phase in the SNX10 OE group at 72 hours in the HPAF-II cells, but this was not significant in the AsPC1 cells compared with the control. Similar results were also seen in a PDAC line (Panc1), in which SNX10 OE results in reduced proliferation, total cell numbers, and colonies compared with the control group (Supplementary Fig. S3A–S3F). Finally, we determined the effect of OE of SNX10 on the levels of phosphorylation (as a measure of activation) of SRC (nonreceptor tyrosine kinase protein), STAT3, and ERK (mitogen-activated protein kinase), molecules known to promote or be downstream of oncogenic KRAS activity (30–33). All these molecules have significantly decreased levels in cells overexpressing SNX10 (Fig. 3A and B). These results support that SNX10 may play a tumor-suppressive role in mutant KRAS PDAC cells.

### SNX10 depletion promotes tumor progression and aggressiveness and reduces survival in PDAC mouse models

To define the impact of SNX10 in the biology of PDAC, we used a P48-driven Cre recombinase to delete *Snx10* specifically in exocrine pancreatic tissues, both in the presence and absence of oncogenic Kras and Tp53 mutations to generate the CS^fl/fl^, KCS^fl/fl^, and KPCS^fl/fl^ mouse models (Supplementary Fig. S4A). All lines used in this study were genotyped through a probe-based assay distinguishing wild-type, heterozygous, and homozygous floxed alleles. Recombination was confirmed in pancreatic tissues by PCR (Supplementary Fig. S4B). Furthermore, we observed that SNX10 expression is significantly reduced in the CS^fl/fl^, KCS^fl/fl^, and KPCS^fl/fl^ groups compared with the P48 Cre (control mice), KC, and KPC mice, respectively (Supplementary Fig. S4C). Moreover, we measured SNX10 expression at the protein level and found that SNX10 expression significantly declined in the CS^fl/fl^, KCS^fl/fl^, and KPCS^fl/fl^ groups, whereas it significantly reduced in the KPC group compared with control mice (P48 Cre; Supplementary Fig. S4D).

**Figure 4:**
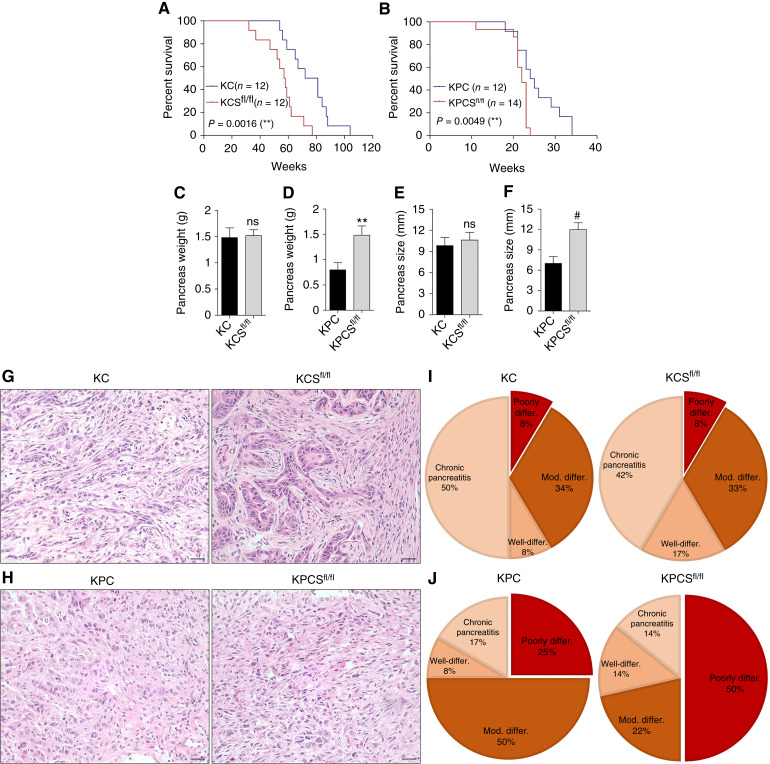
Loss of Snx10 leads to adverse outcomes in PDAC. Kaplan–Meier curves of (**A**) KC (*n* = 12) and KCS^fl/fl^ (*n* = 12) group (*P* = 0.0016). **B,** KPC (*n* = 12) and KPCS^fl/fl^ (*n* = 14) group (*P* = 0.0049). Survival analyses were performed using the log-rank (Mantel–Cox) test. **C** and **D,** Pancreas weight measured in KC (*n* = 12), KCS^fl/fl^ (*n* = 12), KPC (*n* = 12), and KPCS^fl/fl^ (*n* = 14). **E** and **F,** Pancreas size measured in KC (*n* = 12), KCS^fl/fl^ (*n* = 12), KPC (*n* = 12), and KPCS^fl/fl^ (*n* = 14). **G** and **H,** Representative hematoxylin and eosin stains of KC (*n* = 12), KCS^fl/fl^ (*n* = 12), KPC (*n* = 12), and KPCS^fl/fl^ (*n* = 14). Scale bar, 20 μm. **I** and **J,** Pie charts represent the percentage incidence of poorly differentiated, moderately differentiated, chronic pancreatitis, and well-differentiated in KC (*n* = 12), KCS^fl/fl^ (*n* = 12), KPC (*n* = 12), and KPCS^fl/fl^ (*n* = 14). Statistically significant differences *, *P* < 0.05; **, *P* < 0.01; and #, *P* < 0.001 are represented as mean ± SEM.

Analysis of the crosses revealed that the CS^fl/fl^ group does not affect the survival of mice, *P* = 0.1672 (Supplementary Fig. S4E), and showed a normal pancreas based on hematoxylin and eosin staining (Supplementary Fig. S4F). In KPCS^fl/fl^ (*P* = 0.0049) and KCS^fl/fl^ (*P* = 0.0016) mice, the decreased expression of SNX10 lowers survival compared with KPC and KC animals, respectively (Fig. 4A and B). In addition, there was a significant increment in the pancreas weight and size of the KPCS^fl/fl^ group compared with KPC (Fig. 4D and F); however, there was no significant difference in the pancreas weight and size of the KCS^fl/fl^ group compared with KC (Fig. 4C and E). Comprehensive necropsy (isolated pancreas) of all PDAC mouse models revealed more aggressive disease in the KPCS^fl/fl^ mice but not in the KCS^fl/fl^ mice compared with KPC and KC, respectively. Our gross observation of CS^fl/fl^ mice indicated a normal pancreas. In contrast, we observed more abnormalities/tumors in the pancreas of the KPCS^fl/fl^ mice and KCS^fl/fl^ compared with the KPC and KC mice (Supplementary Table S3), which was further confirmed by histopathology analysis.

**Figure 5:**
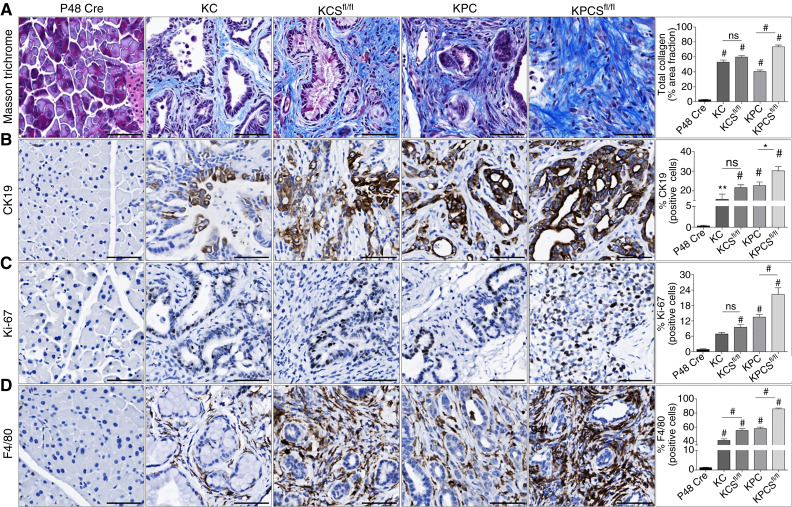
Depletion of Snx10 enhances malignancy markers in PDAC mice: **A–D,** Number of mice per group: P48 Cre (control mouse, *n* = 4), KPC (*n* = 4), KPCS^fl/fl^ (*n* = 4), KC (*n* = 4), and KCS^fl/fl^ (*n* = 4; PDAC mouse). All scale bars, 100 μm. **A,** Masson’s trichrome staining representative (left) images and quantification (right) of total collagen (percentage of area fraction; blue signal). **B,** Representative IHC images (left) and quantification (right) for the ductal marker CK19 and percentage of positive cells (brown signal). **C,** Representative IHC images (left) and quantification (right) for the cell proliferation marker Ki-67 and percentage of positive cells (brown signal). **D,** Representative IHC images (left) and quantification (right) for the macrophage marker F4/80 and percentage of positive cells (brown signal). Significance levels *, *P* < 0.05; **, *P* < 0.01; and #, *P* < 0.001 are represented as mean ± SEM.

Next, we performed hematoxylin and eosin staining of pancreas tissues from the KC, KCS^fl/fl^, KPC, and KPCS^fl/fl^ groups of all survival mice (Fig. 4G and H) and analyzed them based on cancer grades. Fifty percent of KPCS^fl/fl^ animals developed poorly differentiated adenocarcinoma, while only 8% of KCS^fl/fl^ animals developed poorly differentiated adenocarcinoma compared with KPC and KC, respectively. The overall tumor occurrence was higher in KPCS (86%) compared with KPC (83%), as depicted in the pie chart (Fig. 4I and J) with detailed pathology analysis (Supplementary Table S4). We also examined common malignancy markers such as the ductal marker CK19, the proliferation marker Ki-67, the macrophage marker F4/80 to assess activated macrophages/inflammatory conditions, and the collagen fiber marker Masson’s trichrome staining during PDAC progression. We found that the expression of collagen fibers was significantly increased in the KPCS^fl/fl^ mice compared with KPC, whereas the KCS^fl/fl^ mice had no significant difference compared with KC (Fig. 5A). Furthermore, IHC of CK19 expression showed significantly higher expression in the KPCS^fl/fl^ compared with KPC, but there was no significant difference in KCS^fl/fl^ compared with KC (Fig. 5B). Also, we observed significantly higher Ki-67 expression in the KPCS^fl/fl^ tissue compared with KPC, and KCS^fl/fl^ had no significant difference compared with KC (Fig. 5C). Finally, IHC was performed to determine inflammation/activated macrophages (F4/80) in all genotypes and found significantly increased expression in the KPCS^fl/fl^ and KCS^fl/fl^ groups compared with KPC and KC, respectively (Fig. 5D). Furthermore, similar to *in vitro* findings, we found a substantially increased activation of ERK and STAT3 in KPCS^fl/fl^ mice compared with KPC (Supplementary Fig. S5A and S5B). Altogether, our results demonstrated that the loss of SNX10 significantly reduced mice survival, modified the PDAC tumor microenvironment, and promoted tumor proliferation, thus further supporting the tumor suppressor function of this molecule in PDAC biology.

## Discussion

SNX family proteins are generally involved in intracellular trafficking (34); their roles in cancer remain complex. Some members, such as SNX1, SNX6, and SNX27, have oncogenic properties, promoting proliferation and metastasis by activating the TGF-β signaling pathway and recycling cancer-associated proteins in gastric, pancreatic, and colon cancer, respectively (35–37). In contrast, SNX10 has been implicated as a tumor suppressor in gastric, colorectal, and liver cancers (11, 12, 14, 16–18) though its role in PDAC has remained unexplored. This study identifies SNX10 as a novel tumor suppressor candidate in PDAC. Our findings reveal that mutations in SNX10 occur in a significant proportion of PDAC tumors (0.5%–11.7%), with many cooccurring with KRAS gain-of-function mutations. KRAS is a well-established oncogene in PDAC, with mutations present in nearly 90% of cases (32, 38–40), driving tumorigenesis through activation of downstream signaling pathways such as MAPK, mTOR, and Ral (32, 33). SNX10 plays a crucial role in endosomal trafficking (34), a process known to regulate KRAS signaling (41). We observed that SNX10 expression is significantly downregulated in PDAC tissues compared with adjacent normal tissue, at both mRNA and protein levels. Given that alterations in other key tumor suppressors (CDKN2A, SMAD4, and TP53) contribute to PDAC progression (20, 42–45), our data suggest that SNX10 loss may act as a secondary or tertiary change in PDAC pathogenesis.

Previous studies indicated that SNX10 exerts tumor-suppressive effects by regulating autophagy through modulating lysosomal degradation pathways via cargo-mediated autophagy in colorectal cancer (18). Autophagy plays a dual role in pancreatic cancer by supporting tumor cell survival under stress and resistance against therapy (46, 47). Our current data do not address autophagic activity directly. We observed that SNX10 OE in human PDAC cells significantly reduced cell proliferation, tumor initiation, and protumorigenic protein activity. It also induced G_1_-phase cell-cycle arrest, further confirming its role in inhibiting tumor growth. Notably, SNX10 downregulates SRC activity, a key mediator of oncogenic signaling that interacts with receptor tyrosine kinases and G protein–coupled receptors, ultimately activating MAPK, STAT3, and mTOR pathways (12, 14, 18). Given that SRC activation is linked to KRAS mutations and PDAC progression (48–50), our findings suggest that SNX10 loss enhances oncogenic signaling through deregulated SRC activity.

Using a *Snx10* knockout PDAC mouse model, we observed significantly increased tumorigenesis, tumor size, and aggressiveness, particularly in KPCS^fl/fl^ mice. Previously reported PDAC mouse tissues exhibited greater tumor burden, enhanced CK19 expression (a ductal marker), and increased cell proliferation (Ki-67) and macrophage infiltration (F4/80+ tumor-associated macrophages), indicating a more aggressive tumor microenvironment compared with control mouse tissues (51–53). Notably, although KPCS^fl/fl^ mice exhibited significantly worse outcomes and a higher incidence of disease compared with KPC mice, KCS^fl/fl^ mice (lacking Snx10 but without the full KPC background) showed reduced survival but did not develop significantly larger tumors, suggesting that SNX10 loss alone is insufficient to drive aggressive PDAC. IHC analysis further confirmed that SNX10 depletion enhances desmoplasia, inflammation, and tumor proliferation. We observed increased collagen fiber deposition, heightened CK19 and Ki-67 expression, and a significant increase in tumor-associated macrophages in KPCS^fl/fl^ mice compared with KPC controls. These findings indicated a more aggressive tumor microenvironment and increased stromal remodeling in the absence of Snx10. Additionally, the loss of SNX10 led to upregulation of key protumorigenic proteins, including ERK and STAT3. Interestingly, although phosphorylated ERK and STAT3 levels were significantly increased, only moderate changes were observed in total ERK and STAT3. This suggests that SNX10 loss contributes to PDAC progression primarily by activating the ERK and STAT3 pathways, major drivers of cell proliferation and survival in pancreatic cancer.

Our findings establish SNX10’s antitumor function in PDAC, with potential implications for treatment. Given that SNX10 expression is significantly reduced in PDAC tissues, it may serve as a potential therapeutic target. SNX10’s previously reported role in regulating autophagy suggests that it may similarly affect autophagic pathways in pancreatic cancer and contribute to tumor progression. However, further research is needed to elucidate this potential mechanistic connection in PDAC. Future studies should focus on investigating SNX10 expression and its correlation with clinical outcomes in samples from patients with PDAC as well as to identify its direct molecular targets and determining whether restoring SNX10 function can effectively inhibit tumor progression. It is important to evaluate how SNX10 loss influences PDAC metastasis. Although our current study focused primarily on macrophage-driven changes in the tumor microenvironment, the potential role of SNX10 in regulating cancer-associated fibroblasts and broader stromal remodeling permits further study. Although our findings suggest an interplay between SNX10 loss and activation of the SRC/ERK/STAT3 signaling pathways, this relationship remains unclear. Future studies are needed to determine the mechanism underlying this correlation, and whether pharmacologic targeting of the SRC, ERK, or STAT3 pathways can modulate the consequences of SNX10 loss may provide insights into potential therapeutic strategies for PDAC.

### Conclusion

This study establishes SNX10 as a tumor suppressor candidate in PDAC, with its loss contributing to increased tumor growth, aggressiveness, and reduced survival. These findings highlight SNX10 as a potential therapeutic target. Further research on SNX10’s regulatory mechanisms may pave the way for novel treatment strategies aimed at improving patient outcomes in this highly lethal disease.

## Supplementary Material

Supplementary Data Supp Fig 1

Supplementary Data Supp Fig 2

Supplementary Data Supp Fig 3

Supplementary Data Supp Fig 4

Supplementary Data Supp Fig 5

Supplementary Data Supp Table 1

Supplementary Data Supp Table 2

Supplementary Data Supp Table 3

Supplementary Data Supp Table 4
