# Supplementary material for: Loss of Sorting Nexin 10 Accelerates KRAS-Induced Pancreatic Tumorigenesis
Source: Cancer Res Commun. 2025 Sep 8;5(9):1541–51. doi: 10.1158/2767-9764.CRC-25-0168 (PMC12415682; doi:10.1158/2767-9764.CRC-25-0168)
Supplement: Supplementary Data — Supp Fig 1 [file crc-25-0168_supplementary_data_suppsf1.docx]

**Supplementary Figure S1**

**
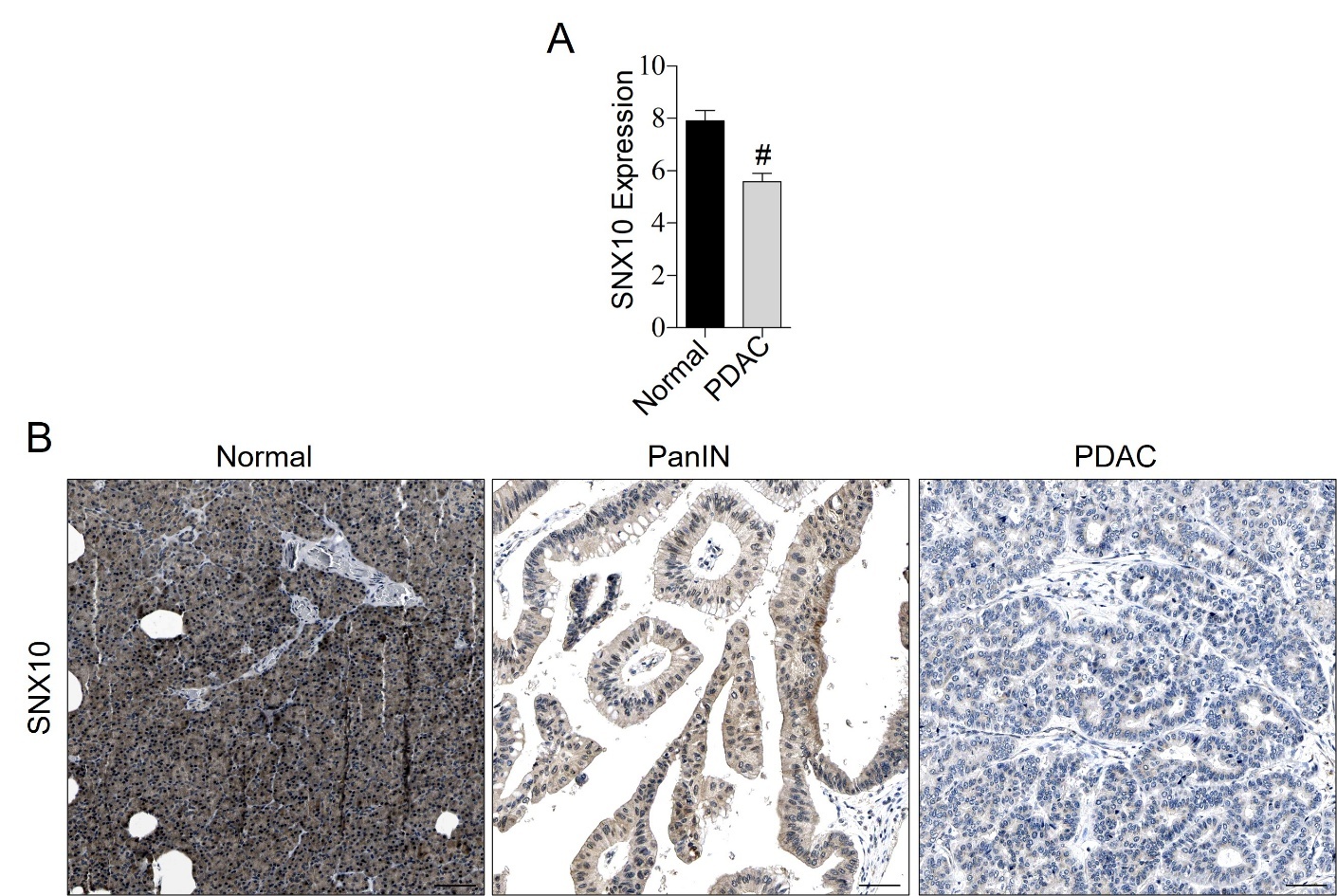
**

**Supplementary Figure S1: SNX10 protein expression is reduced in human PDAC samples (A)** RNA-Seq data from the Human Protein Atlas, including PDAC samples (TCGA dataset, n= 176) [https://www.proteinatlas.org/ENSG00000086300- SNX10/cancer/pancreatic+cancer#PAAD_TCGA](https://www.proteinatlas.org/ENSG00000086300-%20%20SNX10/cancer/pancreatic+cancer#PAAD_TCGA) and adjacent normal control tissues (GTEx dataset, n=328) <https://www.proteinatlas.org/ENSG00000086300-SNX10/tissue/pancreas> showed a significant reduction in SNX10 expression p <0.001 (#) represented mean ± Standard error mean (SEM) **(B)** Representative IHC images of SNX10 (brown signal) procured from the Human Protein Atlas database from adjacent normal control tissues, PanIN and, PDAC <https://www.proteinatlas.org/ENSG00000086300-SNX10/cancer/pancreatic+cancer#IHC>. Scale bar, 100 µm.
