# Supplementary material for: Loss of Sorting Nexin 10 Accelerates KRAS-Induced Pancreatic Tumorigenesis
Source: Cancer Res Commun. 2025 Sep 8;5(9):1541–51. doi: 10.1158/2767-9764.CRC-25-0168 (PMC12415682; doi:10.1158/2767-9764.CRC-25-0168)
Supplement: Supplementary Data — Supp Fig 2 [file crc-25-0168_supplementary_data_suppsf2.docx]

**Supplementary Figure S2**

**
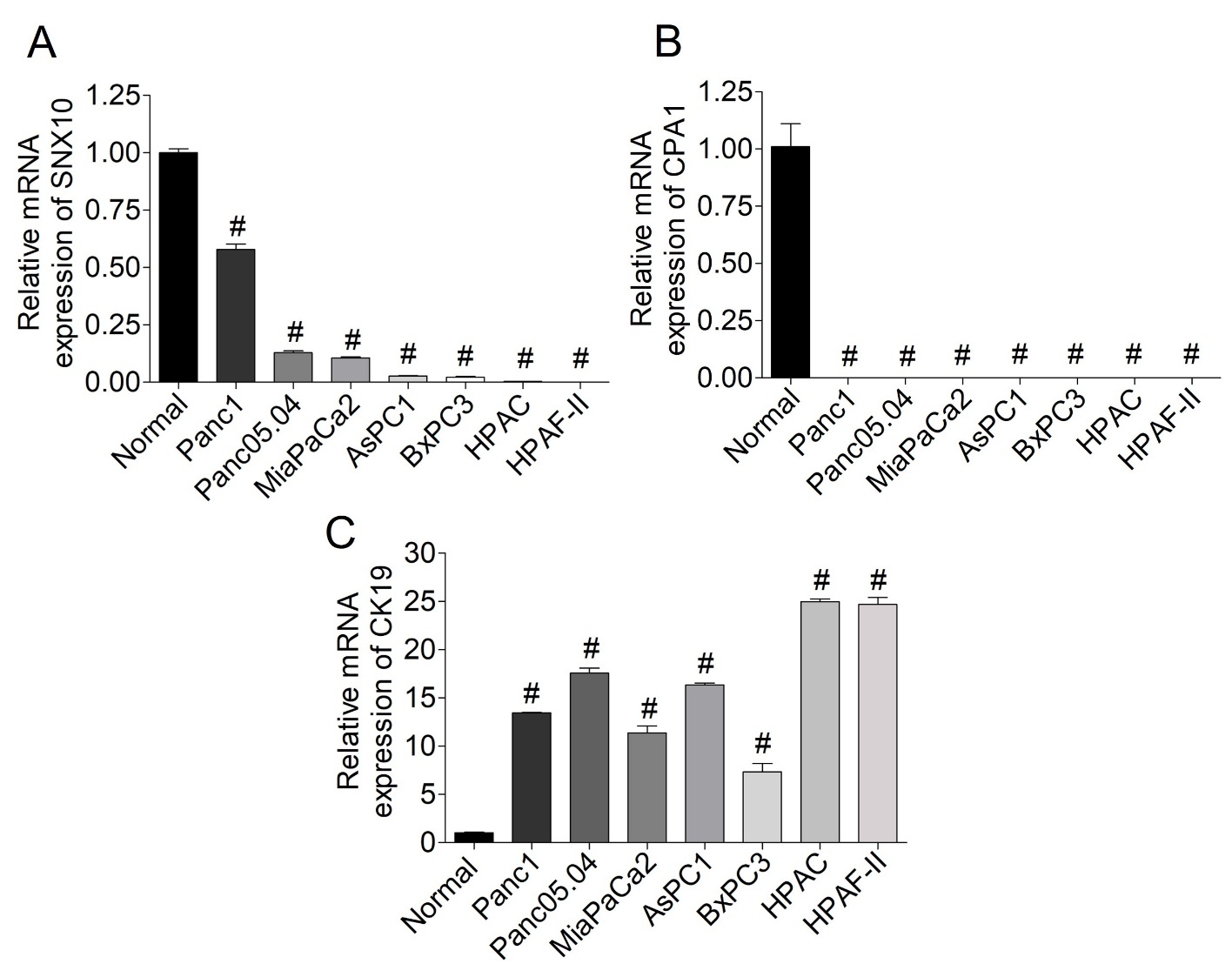
**

**Supplementary Figure S2: SNX10 expression at the mRNA level in PDAC cell lines. (A)** SNX10, **(B)** CPA1, and **(C)** CK19 mRNA expression of the Panc1, Panc05.04, MiaPaCa2, AsPC1, BXPC3, HPAC, and HPAF-II normalized with actin as a reference gene. All PDAC cell lines were compared with the normal human pancreas (RNA). Statistically significant differences P<0.05 (*), P<0.01(**), and P<0.001(#) represented mean ± Standard error mean (SEM) between three independent experiments.
