# Supplementary material for: Loss of Sorting Nexin 10 Accelerates KRAS-Induced Pancreatic Tumorigenesis
Source: Cancer Res Commun. 2025 Sep 8;5(9):1541–51. doi: 10.1158/2767-9764.CRC-25-0168 (PMC12415682; doi:10.1158/2767-9764.CRC-25-0168)
Supplement: Supplementary Data — Supp Fig 3 [file crc-25-0168_supplementary_data_suppsf3.docx]

**Supplementary Figure S3**

**
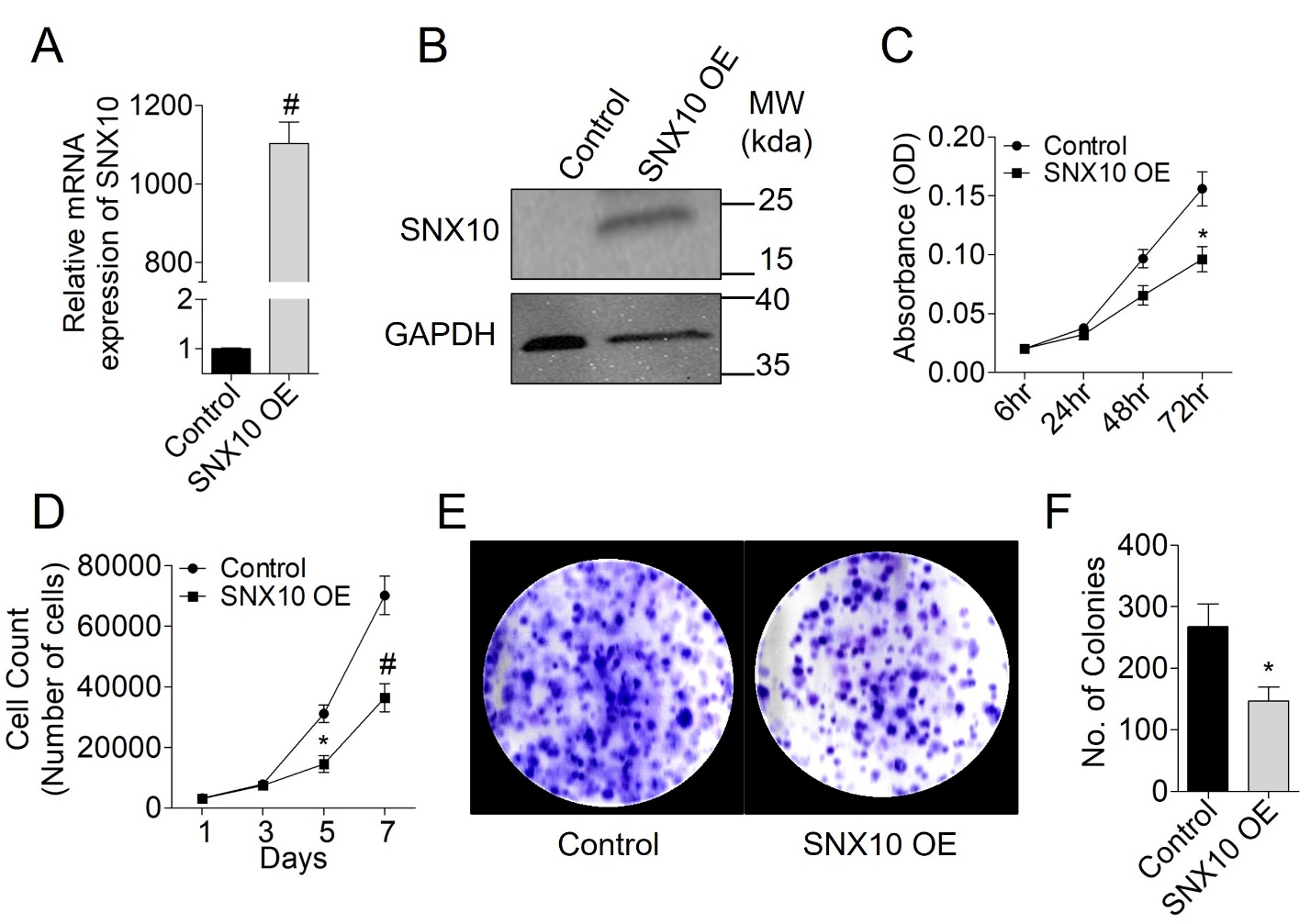
**

**Supplementary Figure S3: Overexpression of SNX10 decreased cell proliferation in the Panc1 PDAC cell line. (A)** SNX10 transfection confirmation in the panc1 cell line by qPCR fold change expression. Actin was used as a reference gene. **(B)** Western blot of control vs. SNX10 OE with GAPDH was used as a loading control for the Panc1 cell line. **(C)** Cell proliferation via MTT (absorbance at 570nm) of Panc1 cells over 72h. **(D)** Growth curve assay of the Panc1 cell line determined the growth rate over a 7-day period. **(E)** Representative images of control vs SNX10 OE colony-forming ability in Panc1 cells. **(F)** Quantification of colony numbers in each group is seen in panel E. Statistically significant differences, P<0.05 (*), P<0.01(**), and P<0.001(#) are represented as mean ± Standard error mean (SEM). SNX10 OE was compared to the control group.
