# Supplementary material for: Loss of Sorting Nexin 10 Accelerates KRAS-Induced Pancreatic Tumorigenesis
Source: Cancer Res Commun. 2025 Sep 8;5(9):1541–51. doi: 10.1158/2767-9764.CRC-25-0168 (PMC12415682; doi:10.1158/2767-9764.CRC-25-0168)
Supplement: Supplementary Data — Supp Fig 4 [file crc-25-0168_supplementary_data_suppsf4.docx]

**Supplementary Figure S4**

**
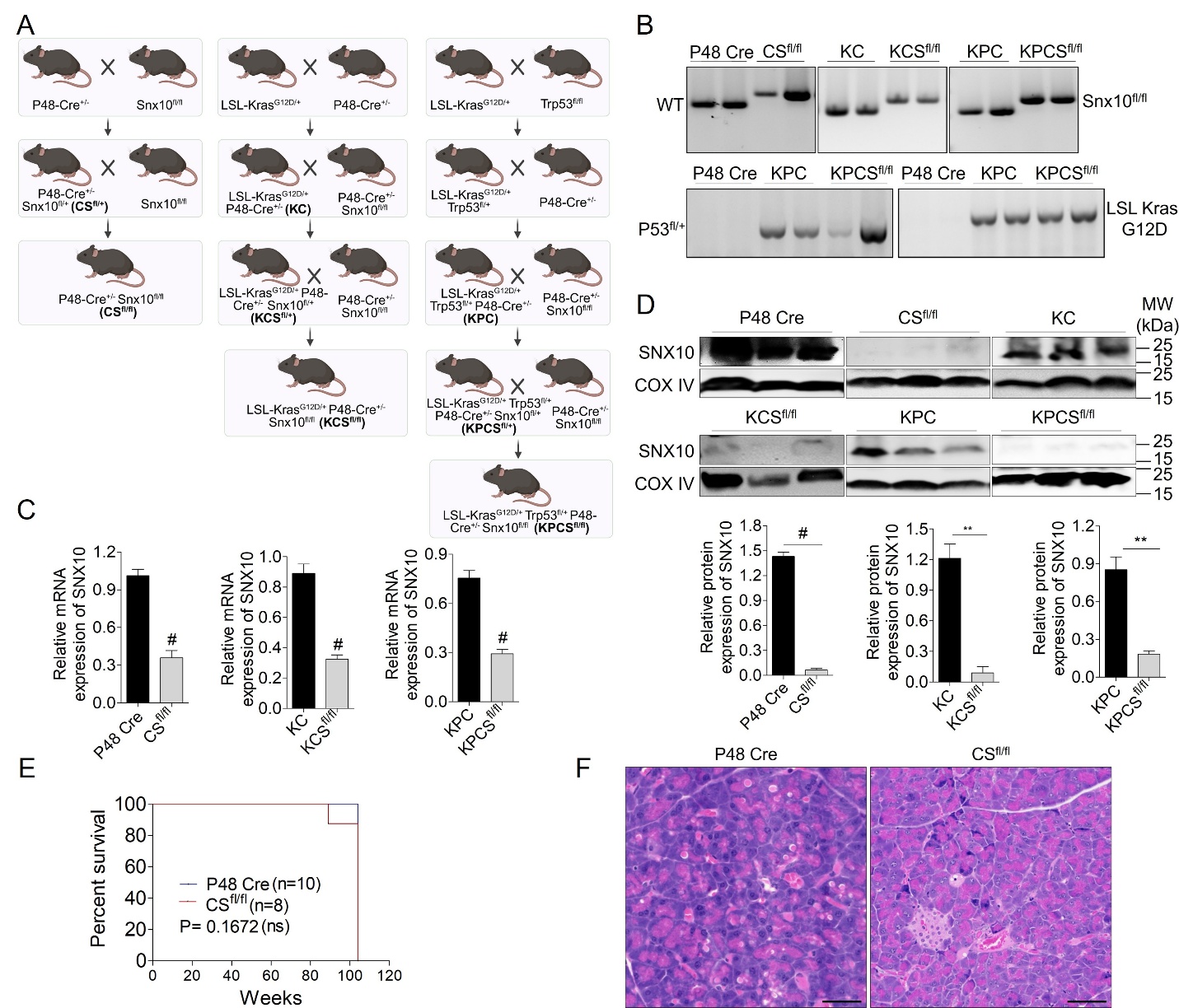
**

**Supplementary Figure S4: Generation and expression of PDAC mouse models. (A)** Final breeding scheme showing generation of CS^fl/fl^, KCS^fl/fl^, and KPCS^fl/fl^ mouse models having mixed genetic backgrounds (FVB and C57BL/6). **(B)** Recombination was confirmed in all experimental animals by PCR analysis of genomic DNA extracted from the pancreas. Images are representative examples from two mice of all the mice included in the study. **(C)** SNX10 fold change expression in P48 Cre, CS^fl/fl^, KC, KCS^fl/fl^, KPC, and KPCS^fl/fl^ models. RPL 13a is used as a reference gene**. (D)** The western blot (Up) of SNX10 expression in the P48 Cre, CS^fl/fl^, KC, KCS^fl/fl^, KPC, and KPCS^fl/fl^ models. Quantification (Down) of relative protein expression of genes in each group from the panel D western blot with COX IV used as a loading control. Data representative of three independent experiments. These are representative blots from three mice of all the mice. All groups compared with P48 Cre, KCS^fl/fl^ compared with KC, and KPCS^fl/fl^ compared with KPC **(E)** Survival curves of P48 Cre (n=10), CS^fl/fl^ (n=8). Survival analyses were performed using the log-rank (Mantel-Cox) test, *P* = 0.1672. **(F)** Representative H&E stain of P48 Cre (n=10) and CS^fl/fl^ (n=8). Scale bar, 20µm. Significance level P<0.05(*), P < 0.01 (**), and P<0.001(#) represented mean ± Standard error mean (SEM).
