# Supplementary material for: Loss of Sorting Nexin 10 Accelerates KRAS-Induced Pancreatic Tumorigenesis
Source: Cancer Res Commun. 2025 Sep 8;5(9):1541–51. doi: 10.1158/2767-9764.CRC-25-0168 (PMC12415682; doi:10.1158/2767-9764.CRC-25-0168)
Supplement: Supplementary Data — Supp Fig 5 [file crc-25-0168_supplementary_data_suppsf5.docx]

**Supplementary Figure S5**

**
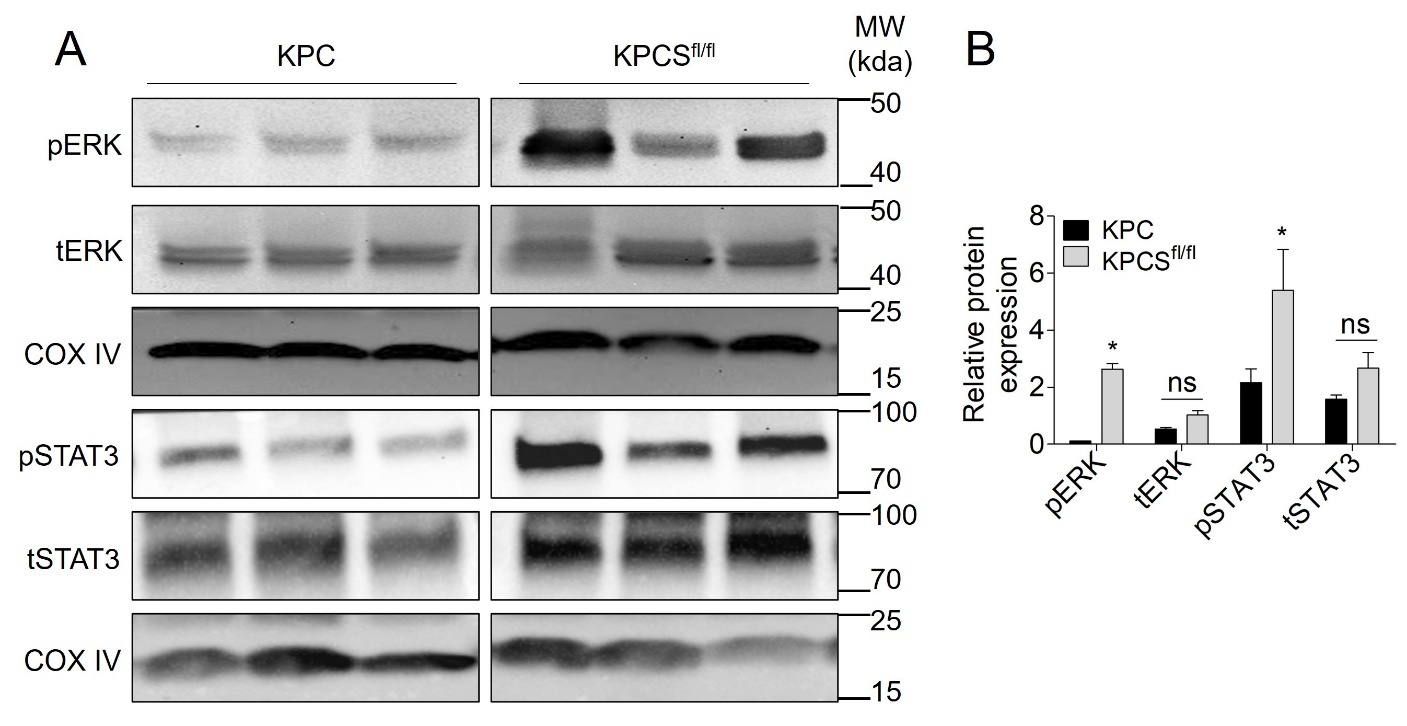
**

**Supplementary Figure S5: Depletion of SNX10 leads to adverse outcomes in PDAC mice. (A)** Western blot showing expression of total and phosphorylation of ERK and STAT3 in KPC (n=12) and KPCS^fl/fl^ (n=14) tissue. COX IV is used as a loading control. These are representative blots from three mice of all the mice. **(B)** Quantification showing the relative protein expression of all genes in each group. Significance level P<0.05 (*), P<0.01(**), and P<0.001(#) represented mean ± Standard error mean (SEM). These are representative figures from one of three independent repeated experiments.
