# Supplementary material for: Loss of Sorting Nexin 10 Accelerates KRAS-Induced Pancreatic Tumorigenesis
Source: Cancer Res Commun. 2025 Sep 8;5(9):1541–51. doi: 10.1158/2767-9764.CRC-25-0168 (PMC12415682; doi:10.1158/2767-9764.CRC-25-0168)
Supplement: Supplementary Data — Supp Table 1 [file crc-25-0168_supplementary_data_suppst1.docx]

**Supplementary Table 1: List of Primers and Antibodies**

| **Name** | **Type** | **Sequence (5’ to 3’)** |
| --- | --- | --- |
| For recombination primers | | |
| LSL-Kras G12D | WT Forward Primer | GTCTTTCCCCAGCACAGTGC |
|  | Mu Forward Primer | AGCTAGCCACCATGGCTTGAGTAAGTCTGCA |
|  | Common Reverse Primer | CTCTTGCCTACGCCACCAGCTC |
| Trp53^fl/+^ | Forward (recombination) | CACAAAAACAGGTTAAACCCAG |
|  | Reverse Primer | GAAGACAGAAAAGGGGAGGG |
| Snx10^fl/fl^ | LoxP3 Forward Primer | ATAACTAACCCAGGCAAACA |
|  | LoxP3 Reverse Primer | TTGTCAAGTGCGTGTGTCGT |

| **Name** | **Company** | **Catalog No.** | **Dilutions** | **RRID** |
| --- | --- | --- | --- | --- |
| Immunofluorescence/ Immunohistochemistry Antibodies | | | | |
| SNX10 | Bioss | bs-12408R-Cy3 | 1:200 | AB_3677590 |
| CPA1 | Bioss | bs-6034R-BF488 | 1:200 | AB_3677591 |
| CK19 | Abcam | Ab52625 | 1:800 | AB_2281020 |
| F4/80 | Cell Signaling Tech | 70076 | 1:1000 | AB_2799771 |
| Ki67 | Abcam | Ab16667 | 1:100 | AB_302459 |

| **Name** | **Company** | **Catalog No.** |
| --- | --- | --- |
| For qPCR (TaqMan gene expression assay) | | |
| β-Actin (human) | Thermofisher Scientific | Hs01060665 |
| SNX10 (human) | Thermofisher Scientific | Hs01007226 |
| CK19 (human) | Thermofisher Scientific | Hs01051611 |
| CPA1 (human) | Thermofisher Scientific | Hs01056157 |
| Snx10 (mouse) | Thermofisher Scientific | Mm00511052 |
| CPA1 (mouse) | Thermofisher Scientific | Mm00465942 |
| RPL 13a (mouse) | Thermofisher Scientific | Mm01612986 |

| **Name** | **Company** | **Catalog No.** | **Dilutions** | **RRID** |
| --- | --- | --- | --- | --- |
| For Western blot (Antibodies) | | | | |
| GAPDH | Cell Signaling Tech | 5174 | 1:1000 | AB_10622025 |
| COX IV | Cell Signaling Tech | 11967 | 1:1000 | AB_2797784 |
| SNX10 (in vitro) | Origene | TA808896 | 1:2000 | AB_3677589 |
| SNX10 (in vivo) | Sigma | SAB2107086 | 1:1000 | AB_3677588 |
| pSTAT3 (Tyr 705) | Cell Signaling Tech | 9145 | 1:2000 | AB_2491009 |
| STAT3 | Cell Signaling Tech | 9139 | 1:1000 | AB_331757 |
| pSRC (Tyr 416) | Cell Signaling Tech | 6943 | 1:1000 | AB_10013641 |
| SRC | Cell Signaling Tech | 2109 | 1:1000 | AB_2106059 |
| pERK (Thr 202/Tyr 204) | Cell Signaling Tech | 4370 | 1:1000 | AB_2315112 |
| ERK | Cell Signaling Tech | 4695 | 1:1000 | AB_390779 |
| Anti-mouse IgG, HRP linked | Cell Signaling Tech | 7076 | 1:3000 | AB_330924 |
| Anti-rabbit IgG, HRP linked | Cell Signaling Tech | 7074 | 1:3000 | AB_2099233 |
