# Supplementary material for: Loss of Sorting Nexin 10 Accelerates KRAS-Induced Pancreatic Tumorigenesis
Source: Cancer Res Commun. 2025 Sep 8;5(9):1541–51. doi: 10.1158/2767-9764.CRC-25-0168 (PMC12415682; doi:10.1158/2767-9764.CRC-25-0168)
Supplement: Supplementary Data — Supp Table 2 [file crc-25-0168_supplementary_data_suppst2.docx]

**Supplementary Table 2: IHC/IF analysis parameters**

| Parameter | Value/Description (QuPath) |
| --- | --- |
| Analysis | Positive cell detection |
| Channel | DAB |
| Threshold | 1 |
| Sigma | 1.5 |
| Detection image | Optical density sum |
| Object classifier trained | Yes (manually annotated to identify positive, negative, and stromal cells) |

| Parameter | Value/Description (ImageJ) |
| --- | --- |
| Analysis | Mean fluorescence intensity |
| Minimum Threshold | 30 |
| Maximum Threshold | 255 |
