# Supplementary material for: Loss of Sorting Nexin 10 Accelerates KRAS-Induced Pancreatic Tumorigenesis
Source: Cancer Res Commun. 2025 Sep 8;5(9):1541–51. doi: 10.1158/2767-9764.CRC-25-0168 (PMC12415682; doi:10.1158/2767-9764.CRC-25-0168)
Supplement: Supplementary Data — Supp Table 3 [file crc-25-0168_supplementary_data_suppst3.docx]

**Supplementary Table 3: Gross observation of disease in the PDAC mouse model**

| **No.** | **ID** | **Genotype** | **Gender** | **DOB** | **DOD** | **Age (weeks)** | **Pancreas** |
| --- | --- | --- | --- | --- | --- | --- | --- |
| 1 | 3844 | P48 Cre | M | 2/3/22 | 1/31/24 | 104 | N |
| 2 | 4129 | P48 Cre | M | 3/17/22 | 3/7/24 | 104 | N |
| 3 | 4092 | P48 Cre | M | 3/13/22 | 1/22/24 | 104 | N |
| 4 | 4305 | P48 Cre | M | 4/8/22 | 4/10/24 | 105 | N |
| 5 | 4308 | P48 Cre | M | 4/8/22 | 4/10/24 | 105 | N |
| 6 | 4088 | P48 Cre | F | 3/13/22 | 3/7/24 | 104 | N * |
| 7 | 4125 | P48 Cre | F | 3/17/22 | 3/7/24 | 104 | N |
| 8 | 4126 | P48 Cre | F | 3/17/22 | 3/7/24 | 104 | N |
| 9 | 4469 | P48 Cre | F | 5/3/22 | 5/6/24 | 105 | N |
| 10 | 4473 | P48 Cre | M | 5/3/22 | 5/6/24 | 105 | N |
| 11 | 4787 | CS^fl/fl^ | M | 6/5/22 | 5/31/24 | 104 | N |
| 12 | 4751 | CS^fl/fl^ | F | 6/1/22 | 5/31/24 | 104 | N |
| 13 | 4717 | CS^fl/fl^ | M | 5/31/22 | 5/31/24 | 104 | N |
| 14 | 4833 | CS^fl/fl^ | M | 6/9/22 | 6/6/24 | 104 | N |
| 15 | 4984 | CS^fl/fl^ | F | 6/27/22 | 6/25/24 | 104 | N |
| 16 | 5123 | CS^fl/fl^ | F | 7/14/22 | 7/11/24 | 104 | N |
| 17 | 4784 | CS^fl/fl^ | F | 6/5/22 | 2/20/24 | 89 | N |
| 18 | 4273 | CS^fl/fl^ | M | 4/6/22 | 4/5/24 | 104 | N |
| 19 | 4053 | KC | M | 3/9/22 | 4/26/23 | 59 | Y |
| 20 | 4382 | KC | M | 4/20/22 | 5/2/23 | 54 | Y |
| 21 | 4658 | KC | M | 5/20/22 | 8/29/23 | 67 | Y |
| 22 | 4369 | KC | M | 4/15/22 | 9/1/23 | 72 | Y |
| 23 | 4010 | KC | M | 2/28/22 | 10/11/23 | 84 | Y |
| 24 | 4379 | KC | M | 4/20/22 | 12/22/23 | 87 | Y |
| 25 | 4226 | KC | F | 3/30/22 | 6/28/23 | 65 | Y |
| 26 | 4774 | KC | F | 6/4/22 | 6/28/23 | 56 | Y |
| 27 | 3894 | KC | F | 2/16/22 | 9/8/23 | 81 | Y |
| 28 | 4290 | KC | F | 4/8/22 | 12/12/23 | 88 | Y |
| 29 | 4777 | KC | F | 6/4/22 | 12/22/23 | 81 | Y |
| 30 | 3893 | KC | F | 2/16/22 | 2/15/24 | 104 | Y |
| 31 | 3575 | KCS^fl/fl^ | M | 12/28/21 | 2/8/23 | 58 | Y |
| 32 | 4602 | KCS^fl/fl^ | M | 5/16/22 | 7/6/23 | 59 | Y |
| 33 | 5455 | KCS^fl/fl^ | M | 8/30/22 | 11/6/23 | 62 | Y |
| 34 | 7300 | KCS^fl/fl^ | M | 6/23/23 | 2/6/24 | 32 | Y |
| 35 | 5929 | KCS^fl/fl^ | M | 11/7/22 | 3/19/24 | 71 | Y |
| 36 | 4034 | KCS^fl/fl^ | F | 3/2/22 | 4/3/23 | 57 | Y |
| 37 | 5110 | KCS^fl/fl^ | F | 7/12/22 | 6/9/23 | 47 | Y |
| 38 | 4387 | KCS^fl/fl^ | F | 4/20/22 | 6/23/23 | 61 | Y |
| 39 | 5717 | KCS^fl/fl^ | F | 10/10/22 | 10/10/23 | 52 | Y |
| 40 | 6476 | KCS^fl/fl^ | F | 1/30/23 | 10/17/23 | 37 | Y |
| 41 | 5739 | KCS^fl/fl^ | F | 10/17/22 | 10/27/23 | 54 | Y |
| 42 | 4955 | KCS^fl/fl^ | F | 6/24/22 | 12/12/23 | 77 | Y |
| 43 | 4568 | KPC | M | 5/10/22 | 10/25/22 | 24 | Y |
| 44 | 4674 | KPC | M | 5/24/22 | 10/30/22 | 23 | Y |
| 45 | 6162 | KPC | M | 12/8/22 | 5/1/23 | 21 | Y |
| 46 | 7806 | KPC | M | 9/23/23 | 2/20/24 | 21 | Y |
| 47 | 7807 | KPC | M | 9/23/23 | 5/16/24 | 34 | Y |
| 48 | 4461 | KPC | F | 5/3/22 | 12/5/22 | 31 | Y |
| 49 | 4462 | KPC | F | 5/3/22 | 10/10/22 | 23 | Y |
| 50 | 4464 | KPC | F | 5/3/22 | 11/23/22 | 29 | Y |
| 51 | 4669 | KPC | F | 5/24/22 | 11/22/22 | 26 | Y |
| 52 | 4978 | KPC | F | 6/26/22 | 10/30/22 | 18 | Y |
| 53 | 5269 | KPC | F | 8/5/22 | 3/28/23 | 34 | Y |
| 54 | 6696 | KPC | F | 3/6/23 | 8/29/23 | 25 | Y |
| 55 | 4492 | KPCS^fl/fl^ | M | 5/4/22 | 10/10/22 | 23 | Y |
| 56 | 4849 | KPCS^fl/fl^ | M | 6/13/22 | 12/8/22 | 21 | Y |
| 57 | 5163 | KPCS^fl/fl^ | M | 7/21/22 | 12/22/22 | 22 | Y |
| 58 | 5267 | KPCS^fl/fl^ | M | 8/4/22 | 12/22/22 | 20 | Y |
| 59 | 5570 | KPCS^fl/fl^ | M | 9/19/22 | 2/17/23 | 22 | Y |
| 60 | 5737 | KPCS^fl/fl^ | M | 10/11/22 | 3/24/23 | 23 | Y |
| 61 | 4353 | KPCS^fl/fl^ | F | 4/15/22 | 9/23/22 | 23 | Y |
| 62 | 5910 | KPCS^fl/fl^ | F | 11/4/22 | 1/18/23 | 11 | Y |
| 63 | 5264 | KPCS^fl/fl^ | F | 8/4/22 | 1/13/23 | 23 | Y |
| 64 | 5274 | KPCS^fl/fl^ | F | 8/5/22 | 12/29/22 | 21 | Y |
| 65 | 5445 | KPCS^fl/fl^ | F | 8/30/22 | 2/8/23 | 23 | Y |
| 66 | 5942 | KPCS^fl/fl^ | F | 11/9/22 | 4/6/23 | 21 | Y |
| 67 | 5924 | KPCS^fl/fl^ | F | 11/7/22 | 4/24/23 | 24 | Y |
| 68 | 6684 | KPCS^fl/fl^ | F | 3/3/23 | 8/1/23 | 21 | Y |

N normal healthy pancreas, Y abnormal pancreas, *normal but some islet cell hyperplasia
