# Supplementary material for: Loss of Sorting Nexin 10 Accelerates KRAS-Induced Pancreatic Tumorigenesis
Source: Cancer Res Commun. 2025 Sep 8;5(9):1541–51. doi: 10.1158/2767-9764.CRC-25-0168 (PMC12415682; doi:10.1158/2767-9764.CRC-25-0168)
Supplement: Supplementary Data — Supp Table 4 [file crc-25-0168_supplementary_data_suppst4.docx]

**Supplementary Table 4: Histopathology analysis of PDAC mouse models**

| **Mice Group** | **P48 Cre** | **KC** | **KCS^fl/fl^** | **KPC** | **KPCS^fl/fl^** |
| --- | --- | --- | --- | --- | --- |
| Poorly differentiated | 0 | 1 | 1 | 3 | 7 |
| Moderately differentiated | 0 | 4 | 4 | 6 | 3 |
| Well- differentiated | 0 | 1 | 2 | 1 | 2 |
| Chronic pancreatitis | 0 | 6 | 5 | 2 | 2 |
| Total | 10 | 12 | 12 | 12 | 14 |
